# Cancer immunosurveillance by CD8 T cells

**DOI:** 10.12688/f1000research.21150.1

**Published:** 2020-02-03

**Authors:** José C Crispin, George C Tsokos

**Affiliations:** 1 Escuela de Medicina y Ciencias de la Salud, Tecnológico de Monterrey, Mexico City, Mexico; 2 Department of Immunology and Rheumatology, Instituto Nacional de Ciencias Médicas y Nutrición Salvador Zubirán, Mexico City, Mexico; 3 Department of Medicine, Beth Israel Deaconess Medical Center, Boston, USA; 4 Harvard Medical School, Boston, USA

**Keywords:** T cell, CD8, PD-1, exhaustion, cancer

## Abstract

Clinical success attained in patients with cancer treated with checkpoint inhibitors has renewed the interest in the immune system and in particular in T cells as a therapeutic tool to eliminate tumors. Here, we discuss recent studies that evaluate the anti-tumor role of CD8 T cells and the mechanisms that interfere with this function. In particular, we review recent literature that has reported on the phenotype and transcriptome of tumor-infiltrating CD8 T cells and deciphered the mechanisms associated with failed tumor rejection.

The term “immunological surveillance” was coined by Burnet, when he proposed that long-lived animals with multi-cellular systems may use the immune system to deal with the occurrence of somatic mutation and potential neoplasia ^1^. The increased incidence of malignant diseases in immunosuppressed patients and experimental animals supports the importance of immunosurveillance ^2^. However, there is little evidence that immunosurveillance is a physiological function of CD8 T cells. Because they recognize antigens presented by widely expressed class I major histocompatibility complex (MHC) molecules, CD8 T cells have a very high destructive potential that is curbed by central and peripheral mechanisms of immune tolerance ^3^. Accordingly, most malignant cells are not detected by CD8 T cells. Tumors that are recognized by CD8 T cells usually display a high mutational burden, a phenomenon associated with the expression of aberrant molecular signatures ^4– 6^. However, even when primed by antigens expressed by tumors, CD8 T cells often fail to control tumor growth, because they become inactivated in the tumor microenvironment (TME) ^7^. The clinical efficacy of therapies that target some of the inhibitory mechanisms that regulate anti-tumoral CD8 function (for example, PD-1 blocking antibodies) indicates that CD8 T-cell manipulation is promising in the setting of certain types of cancer. Here, we will discuss recent work that studies the behavior and function of CD8 T cells as anti-tumor effectors.

Tumor infiltration by CD8 T cells is associated with better prognosis and response in several types of cancer ^8– 10^, probably because it indicates that tumor-derived antigens have primed a CD8 T-cell anti-tumor response and that activated CD8 T cells have reached the tumor. However, even in this scenario, CD8 T cells are commonly not able to destroy the tumor. Recent studies have analyzed the gene expression profile of intra-tumoral CD8 T cells at a single-cell level in an attempt to understand why CD8 T cells that are primed against tumor antigens and have infiltrated the tumor fail to eliminate it ^11^. Most reports agree that a transcriptional signature of T-cell activation confers good prognosis but that a signature of exhaustion or regulatory T (Treg) cells is indicative of worse prognosis ^12– 15^. In this context, *exhaustion* refers to a T-cell phenotype first described in CD8 T cells exposed to a chronic viral infection ^16, 17^. Virus-specific CD8 T cells found in individuals with chronic viral infections, that fail to demonstrate effector capacities (that is, proliferation, cytokine production, and cytotoxicity) when activated through the T-cell receptor (TCR), are *exhausted*. This functional inactivation has been associated with the expression of a variety of co-inhibitory molecules, in particular PD-1, and with epigenetic and metabolic reprogramming (reviewed in 18). Intra-tumoral CD8 T cells share phenotypic and transcriptomic features with exhausted T cells found in chronic viral infections. Accordingly, it has been proposed that chronic antigenic stimulation, in the context of viral infections or tumors, induces T-cell exhaustion and that this process impedes anti-tumoral CD8 T-cell responses.

Studies that have analyzed the tumor-infiltrating T-cell transcriptome at a single-cell level have found that tumor-infiltrating lymphocytes (TILs) can be segregated into clusters according to their gene expression profile. Although the clusters vary in each analysis, the studies agree that tumors rich in T cells that display a gene profile indicative of exhaustion exhibit a worse clinical response ^13, 15, 19^. This agrees with the concept that TILs that become exhausted in the TME fail to exert anti-tumor activities. The aim of anti-PD-1 therapy is to *reinvigorate* these cells so they recover their effector functions ^20^.

The exhaustion signature found in TILs is complex and variable, and some studies have tried to identify key elements of this gene profile to use them as biomarkers or to propose them as therapeutic targets. *TCF7* (previously known as TCF-1), a transcription factor essential for differentiation and persistence of memory CD8 T cells ^21^, was identified as a main component of the gene signature found in responding melanomas ^13^. Melanomas rich in TCF7 responded better and showed a longer overall survival rate than melanomas with lower expression of TCF7 ^13^. Paradoxically, TCF7 has been linked to T-cell exhaustion ^18^. However, recent reports indicate that TCF7 is present in *early* exhausted T cells, which are the cells that can be reinvigorated by PD-1 blockade, in contrast to *terminally* exhausted T cells that no longer express TCF7 and are refractory to anti-PD-1 treatment ^22, 23^. This concept is supported by work that has shown that TCF7 marks intra-tumoral CD8 T cells with stem-like properties ^24, 25^ that represent a self-renewing pool of tumor-specific T cells that gives rise to terminally differentiated cells, particularly after checkpoint blockade ^10^. Thus, TCF7-positive T cells are tumor-specific CD8 cells that express PD-1 and other exhaustion-associated markers as a result of chronic activation but are able to functionally recover in response to PD-1 inhibition. Therefore, the larger the fraction of TCF7 cells, the better the response to immunotherapy ( Figure 1).

**Figure 1.**
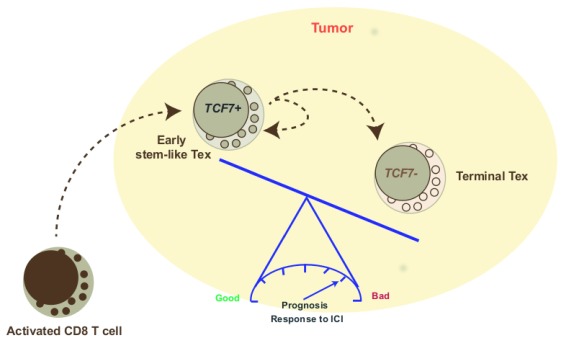
CD8 T-cell exhaustion in tumors determines prognosis and response to treatment. CD8 T cells primed by tumor-derived antigens acquire effector functions and migrate to the tumor. The tumor microenvironment induces T-cell exhaustion through complex and not completely understood mechanisms that include repetitive antigenic stimulation, expression of co-inhibitory molecules (for example, PD-L1), abundance of inhibitory soluble molecules (for example, prostaglandin E2, adenosine, transforming growth factor beta, and interleukin-10), and regulatory T cells. Early exhausted T cells (Early stem-like Tex) express intermediate levels of PD-1 and the transcription factor TCF7 (TCF-1) that grants them self-renewing properties. Anti-PD-1 therapy is able to reinvigorate this population and, in some tumors, its abundance predicts good response to PD-1 blockade. Terminally exhausted T cells (Terminal Tex) no longer express TCF7 and bear high levels of PD-1. These cells fail to respond to PD-1 blockade but may regain effector capacities when other molecules (for example, TIM3 and CD39) are inhibited. ICI, immune checkpoint inhibitor.

Co-expression of TIM3 and CD39 identified exhausted T cells with a gene expression profile analogous to the one associated with failure to respond to PD-1 blockade. These cells, which did not express TCF7, probably represented terminally exhausted cells. TIM3, encoded by *HAVCR2*, is a marker previously associated with CD8 T-cell exhaustion in patients with chronic viral infections and cancer (melanoma and breast) ^26^. TIM3 ^+^ CD39 ^+^ double-positive cells exhibited defective cytokine production and cytotoxic capacity. Importantly, administration of a CD39 inhibitor plus TIM3 blocking antibodies reduced tumor size and increased survival in a melanoma mouse model ^13^, suggesting that blockade of pathways different from PD-1 may be beneficial, in particular in tumors rich in terminally exhausted CD8 cells ^27^. The identification of additional co-inhibitory (for example, LAG-3) and co-stimulatory (for example, ICOS) molecules that can be blocked or activated to boost anti-tumor immunity is currently an area of intense research ^28, 29^.

*LAYN*, the gene that encodes layilin, was identified as being overexpressed in Treg cells from lung and colon cancer ^30^. A more recent study reported that *LAYN* transcription was high in Treg cells and in exhausted CD8 T cells from hepatocellular carcinoma infiltrates ^19^. High *LAYN* expression in liver cancer predicted a poorer overall survival and forced expression of *LAYN* in CD8 T cells inhibited interferon gamma (IFN-γ) production, suggesting that it may inhibit CD8 T-cell effector functions ^19^. Little is known about its function in Treg cells and, in particular, whether its high expression in intra-tumoral Treg cells promotes their suppressive function ^31^. However, the fact that this gene is expressed by Treg cells and is associated with decreased CD8 T-cell function suggests that it may impair anti-tumor immunity through more than one mechanism.

Analyses of the sequences of the rearranged TCR-α and -β genes in TIL have demonstrated the presence of variable numbers of T-cell clones and have allowed researchers to infer the relationship between clone size and activation state. A recent study looked at TIL in patients with basal cell carcinoma, before and after anti-PD-1 therapy ^15^. Treatment with anti-PD-1 was associated with a significant increase in the abundance of cells displaying a gene profile that suggested chronic activation, T-cell dysfunction, and tumor reactivity ^15^. The authors found that, within clones, the gene signature tended to be similar, suggesting that TCR specificity contributes to CD8 fate within the tumor. Importantly, although PD-1 blockade was followed by a clinical response, increased immune cell tumor infiltration, and tumor immunoediting ^32^, it was not associated with a decrease in TILs with an exhausted signature or with an increase in cells with an effector phenotype. This could be related to how exhaustion is defined in terms of gene signature, but it indicates that, within tumors, CD8 T cells acquire features that suggest exhaustion relatively fast and that CD8 T cells can exert effector functions to a certain degree even in the presence of exhaustion-related genes ^15^. Another interesting finding of that work was that PD-1 blockade led to the expansion of clones that had not been detected in the tumors in the pre-treatment biopsies, suggesting that rather than inducing the reinvigoration of exhausted clones, PD-1 inhibition allowed new anti-tumor clones to become activated and recruited ^15^. This phenomenon, however, has not been documented in other tumors and may represent a feature of basal cell carcinoma ^13, 32^.

CD8 T-cell exhaustion is a mechanism that protects self-tissues from the potentially noxious effects of CD8 T cells. Several factors have been suggested to promote CD8 exhaustion and they probably vary according to tissue and context. However, persistent TCR signaling may be the most important factor that programs a CD8 T cell to become exhausted ^18^. The most expanded clones in anti-PD-1–treated tumors were found within exhausted CD8 T cells, which also displayed high proliferation, suggesting that a stronger anti-tumor response is also a stronger promoter of exhaustion ^15^.

Tumor and organ (that is, skin, kidney, and lung) tissue resident memory (T _RM_) CD8 cells have a gene expression profile which is different from that expressed by CD8 memory cells in the periphery but similar to that described for TIL. A recent study documented the importance of the transcription factor Runx3 in the establishment of T _RM_ and their presence in tissues ^33^. Given that Runx3 has variable actions on various genes in various tissues, more information will be needed to understand its role in inflammatory and autoimmune conditions. The presence of Runx3-expressing T _RM_ in tumors determines the vigor of the anti-tumor response, yet they express high amounts of both inhibitory/exhaustion and activation markers, calling for better understanding of exhaustion markers in the regulation of the immune response.

In the setting of autoimmune disease, the presence of exhaustion signatures in peripheral blood indicates a better prognosis and a lower frequency of disease relapse ^34, 35^. However, as observed in tumors and chronic infections, CD8 T-cell inactivation in response to persistent antigen exposure in the setting of autoimmunity is complex and variable. CD8 T cells exposed to ubiquitous cognate antigen lose the expression of CD8 and upregulate inhibitory molecules, in particular PD-1, without activating a complete exhaustion signature ^36^, and cells bearing this phenotype are present in normal mice during steady state, suggesting that self-reactive CD8 T cells are continuously being checked by this mechanism ^37^.

The available data indicate that CD8 T cells are controlled by complex layers of peripheral tolerance that minimize the risk of inflammatory- and cytotoxicity-mediated tissue damage. Immunosurveillance of tumors probably results from the combined actions of natural killer cells and tissue-resident sentinel cells that detect cellular distress and phenotypic changes and occasionally trigger antigen-specific immune responses. Activated anti-tumor CD8 T cells fail to control tumor growth in patients who develop cancer. However, they linger in the tumor and can be reactivated to different degrees by therapies that block the mechanisms that are keeping them in check. These mechanisms vary between individuals and probably within the same tumor, according to location (for example, periphery versus core) and time. A deeper and more comprehensive understanding of the factors that control CD8 T-cell effector function and of the pathways through which these mechanisms exert their functions will allow us to design better therapeutic strategies to deal with this complex clinical problem.
